# Algerian Olive Germplasm and Its Relationships with the Central-Western Mediterranean Varieties Contributes to Clarify Cultivated Olive Diversification

**DOI:** 10.3390/plants10040678

**Published:** 2021-04-01

**Authors:** Kamel Atrouz, Ratiba Bousba, Francesco Paolo Marra, Annalisa Marchese, Francesca Luisa Conforti, Benedetta Perrone, Hamza Harkat, Amelia Salimonti, Samanta Zelasco

**Affiliations:** 1 Council for Agricultural Research and Economics-Research Centre for Olive, Fruit and Citrus Crops, 87036 Rende, Italy; kamalatrouz@gmail.com (K.A.); amelia.salimonti@crea.gov.it (A.S.); 2 Department of Biology and Plant Ecology, Faculty of Natural Sciences and Life, Frères, Mentouri University, Constantine 25000, Algeria; bousbaratiba@gmail.com (R.B.); hamza_harkat@yahoo.com (H.H.); 3 Department of Architecture, University of Palermo, 90128 Palermo, Italy; francescopaolo.marra@unipa.it; 4 Department of Agricultural, Food and Forest Sciences, University of Palermo, 90128 Palermo, Italy; annalisa.marchese@unipa.it; 5 Department of Pharmacy, Health and Nutritional Sciences, University of Calabria, 87036 Rende, Italy; francescaluisa.conforti@unical.it (F.L.C.); benedetta.perrone90@gmail.com (B.P.)

**Keywords:** Algerian olive germplasm, phylogenesis analysis, biodiversity

## Abstract

Olive tree with its main final product, olive oil, is an important element of Mediterranean history, considered the emblematic fruit of a civilization. Despite its wide diffusion and economic and cultural importance, its evolutionary and phylogenetic history is still difficult to clarify. As part of the Mediterranean basin, Algeria was indicated as a secondary diversification center. However, genetic characterization studies from Maghreb area, are currently underrepresented. In this context, we characterized 119 endemic Algerian accessions by using 12 microsatellite markers with the main goal to evaluate the genetic diversity and population structure. In order to provide new insights about the history of olive diversification events in the Central-Western Mediterranean basin, we included and analyzed a sample of 103 Italian accessions from Sicily and, a set of molecular profiles of cultivars from the Central-Western Mediterranean area. The phylogenetic investigation let us to evaluate genetic relationships among Central-Mediterranean basin olive germplasm, highlight new synonymy cases to support the importance of vegetative propagation in the cultivated olive diffusion and consolidate the hypothesis of more recent admixture events occurrence. This work provided new information about Algerian germplasm biodiversity and contributed to clarify olive diversification process.

## 1. Introduction

The cultivated olive tree, (*Olea europaea* L. sub-sp. *europaea* var. bot. *europaea*) is the symbol of the Mediterranean culture and crucial for the Mediterranean diet [1]. Olive with its main final product, which stands out on our tables and in our kitchens, is also an important element of Mediterranean history, considered the emblematic fruit of a civilization. To put it like the historian Fernand Braudel, “where the olive tree ends, the Mediterranean also ends”. However, currently olive growing diffusion has reached a lot of non-traditional producer countries such as Argentina, Australia, Chile, China, and the United States [2]. Despite a wide diffusion and economic and cultural importance of the tree crop, its evolutionary and phylogenetic history is still difficult to clarify [2,3]. The geological period during which the Olea complex originated and began to evolve is difficult to determine; the absence of certain fossil finds makes it difficult to identify a definite epoch. Palaeobotanical data attest to the occurrence of *Olea* sp. in Europe during the Oligocene–Miocene boundary that could be identified as an ancestor of the oleaster (*Olea europaea* L. sub-sp. *europaea* var. bot. *sylvestris*). Phylogenetic analyses have suggested that the most recent common ancestor of the 6 olive subspecies was present during the Late Miocene or Early Pliocene, about 4.0–8.3 million years ago [4]. Besnard et al. [4] indicated the presence of olive trees as a floristic element of African paleotropical origin. Olive trees colonized the Mediterranean area in the Pliocene period and the diversification into the 6 subspecies (subsp. *cuspidata*, *laperrinei*, *guanchica*, *maroccana*, *cerasiformis*, *europaea*) occurred during the last 3–4 million years, following a series of tectonic and climatic events, thus suggesting a specific evolutionary scenario.

Genetic analysis supported the systematic classification and allowed to individuate the subsp. *europaea* var. bot. *sylvestris* as the most recent ancestral of the olive tree cultivated for the largest number of shared plastid lines [5]. All six subspecies show distinct plastid lineages that are partially shared among this group. For instance, subsp. *laperrinei* maintains a shared line with the cultivated olive tree that is not present in subsp. *sylvestris* [3].

The recent accumulation of palaeobotanical, archeological, historical, and molecular data has contributed to improve knowledge, but further investigations are required to elucidate the processes underlying the primary domestication and subsequent secondary diversification of olives. It is commonly believed that olive domestication occurred in the Near East approximately 6000 years ago. The great civilizations of the eastern Mediterranean, such as the Phoenicians, Greeks and Romans, disseminated this tree crop throughout the Mediterranean basin [5,6,7]. Further, recent genetic studies demonstrated that as many as 90% of current cultivars are characterized by the same chloroplast haplotype lineage [3].

The spreading of the olive culture throughout the Mediterranean Basin by human migrations and commercial exchanges has played a key role in determining the current pattern of olive germplasm diffusion. The Maghreb indicates the western most geographical area of North Africa overlooking the Mediterranean Sea and the Atlantic Ocean; originally it concerned the strip of land between the Atlas Mountains and the Mediterranean Sea (northern Tunisia, Algeria and Morocco); some sources also include Islamic Sicily and al-Andalus namely, the Islamic Spain, the name given by the Arabs to the part of the Iberian Peninsula and of Septimania, in the south of France controlled and governed by them. As part of the Mediterranean basin, specifically referred to Maghreb, with a fairly heterogeneous climate between its different regions, Algeria is distinguished by the biological richness of its ecosystems, and it was indicated as a secondary diversification center of several olive subspecies and cultivars [8]. In fact, several cultivars were found, often carrying the name of the farmer who selected them or of the locality where they were grown. This reflected the large number of cultivated varieties, including old or traditional varieties of the country. These genotypes represent an interesting genetic potential for plant breeding programs. Furthermore, despite their abundance, the genetic resources available in Algeria are not sufficiently exploited to improve their performance. Thus, their integration in the breeding programs needs more efforts both in genetic and phenotypic characterization activities. Several prospecting actions have been carried out and variety catalogues have been elaborated in Algeria [9]. However, Algerian germplasm has been only recently studied at molecular level [1,10,11,12] and just two studies carried out a comparison among a relatively low number of Algerian cultivars with the rest of Mediterranean olive germplasm where hypotheses about olive domestication and migration pathway have been formulated [1,11]. Haddad et al. [1] speculated about the existence of an independent domestication center in the Central Mediterranean area, starting from the observation that Laperrine’s olive tree share its haplotype (E1) with most of the world’s olive varieties, including 67% of the Algerian varieties. Di Rienzo et al. [11], using a predictive model, excluded historical migration events between Algerian population and other populations such those from Italy, Syria and Malta, describing a diffusion of olives from Syria to the Greek area, a secondary independent event of domestication from Syria to Spain and a second migration event occurred in Italy and Malte from the Greek area.

Although these and other recent studies have provided nuclear and chloroplastic genetic profiles for wild olives and cultivars from the central and southern Mediterranean regions, the Algerian germplasm still remains underrepresented [1,10,11,12].

Efficient and robust molecular markers are increasingly needed for improving varieties, establishing a molecular database for tree identification enabling the spread of common methods for large-scale genetic analysis. At present, Simple Sequence Repeats (SSRs) or microsatellites offer the opportunity to evaluate many potentially polymorphic sites and are the markers of choice for olive DNA fingerprinting and cultivar identification [13]. Microsatellites also have advantages over other PCR-based markers, because they are co-dominant and easily reproducible, and have a frequent and random distribution, allowing a wide coverage of the genome [14,15]. The high level of variation detected with microsatellites increases the resolution for genealogy and germplasm genetic diversity studies [16,17].

In this context, we collected 119 endemic Algerian accessions belonging to two different subspecies (*europaea* and *laperrinei*) in the Eastern and Southern Algeria regions, respectively and characterized them by using 12 microsatellite markers with the main goal to evaluate the genetic diversity and population structure of a wider sample of Algerian olive tree. Furthermore, to provide new insights about the history of olive diversification such as bottleneck, migration and other demography events in the Central-Western Mediterranean basin, we included and analyzed 103 samples of Italian accessions from Sicily considering its peculiar geographic position and, a set of molecular profiles from French, Tunisian, Moroccan, and Spanish cultivars representative of Central-Western Mediterranean area from our internal database. The phylogenetic investigation of 461 genotypes let us to evaluate genetic relationships among Central-Mediterranean basin olive germplasm, highlight new synonymy cases to support the importance of vegetative propagation in the cultivated olive diffusion and consolidate the hypothesis of more recent admixture events occurrence.

## 2. Results

### 2.1. Genetic Diversity and Polymorphism of SSR Markers in Algerian Population

One hundred nineteen accessions including 109 cultivars, 8 putative wild or feral olive trees, and 2 individuals belonging to *Olea europaea* subsp. *laperrinei* were fingerprinted using 12 SSR loci. A total of 127 alleles were obtained, with sizes varying from the shortest allele with 100 bp for UDO 15 to the longest allele with 308 bp for OLEST 14 (Table 1). The number of alleles per locus ranged from 6 in the SSR locus OLEST 23 to 16 for the DCA 8 locus, with a mean of 10.6 alleles, revealing a high level of variability in the sample set. The number of effective alleles (Ne) per SSR ranged from 1.46 (OLEST 23) to 8.42 (DCA 18), with a mean of 4.06. The observed (Ho) and expected (He) heterozygosity ranged from 0.275 (UDO 15) to 0.945 (DCA 18) and from 0.67 (EMO 90) to 0.885 (DCA 18), with a mean of 0.72 and 0.77, respectively. In fact a Ho significant deficiency was observed for three loci (UDO 15, UDO 12, and DCA 11) with a corresponding very high positive value for both the inbreeding coefficient (Fis) and the probability of null alleles F (Null). Shannon’s information index (I) showed the lowest value 1.37 at locus EMO 90 and the highest value 2.23 at locus DCA 18. Polymorphic information content (PIC) value ranged for the same markers from 0.621 to 0.869 with an average of 0.74 (Table 1). All the microsatellite loci showed higher value of PIC > 0.5 and could be classified as highly informative.

**Figure 1:**
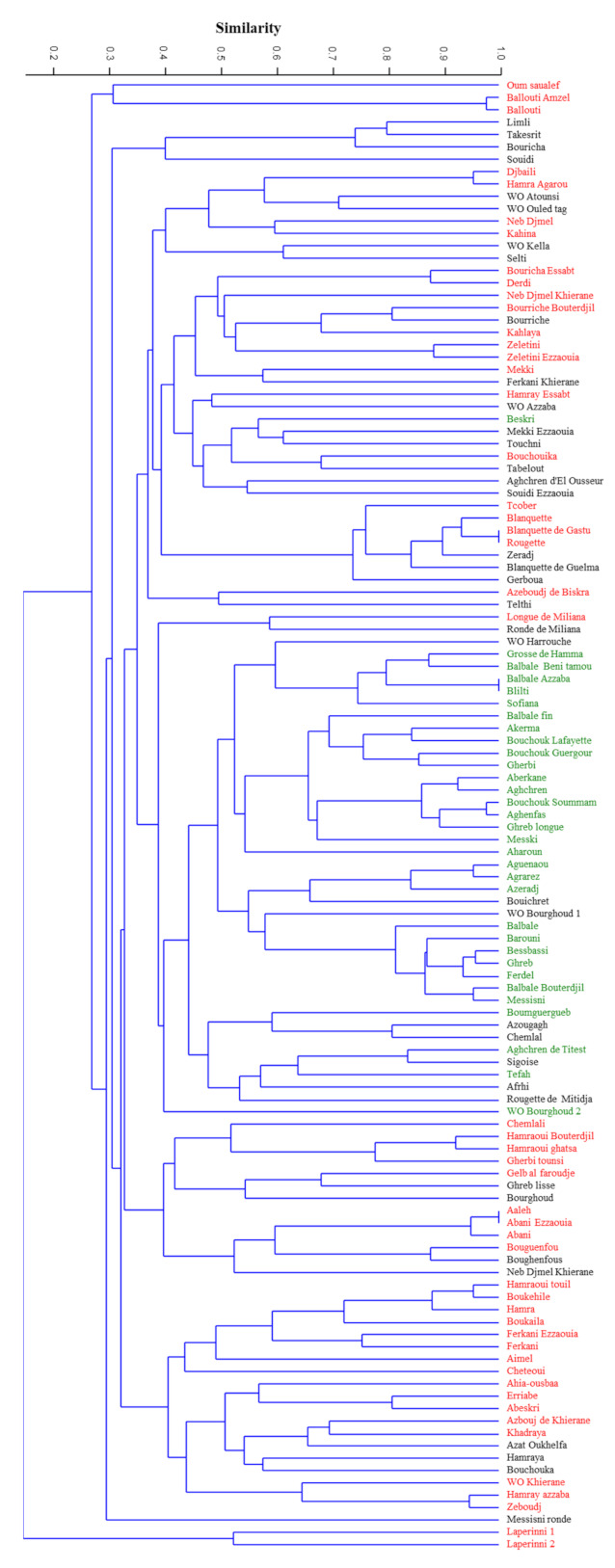
Dendrogram of 119 Algerian olive accessions obtained with 12 SSR markers, based on the UPGMA methodology according to the Dice coefficient. Red and Green colors correspond to the two differentiate groups found by STRUCTURE analysis. Black color refers to admixed genotypes.

The observed frequency of the detected 127 alleles ranged from 0.004 to 0.529, with a mean of 0.0945 (Supplementary Materials Table S1). The highest frequency value (0.529) was shown for the allele 206 bp at locus DCA 8 while 63 rare alleles were detected (frequency < 5%), representing almost 50% of the total of 127 amplified alleles. New alleles were detected for almost all the SSR loci: 137 bp (GAPU 71b), 228 bp (DCA 3), 119 bp and 125 bp (DCA 8), 192 bp and 222 bp (DCA 5), 202 bp (EMO 90), 153 bp and 162 bp (UDO 12), 109 bp and 113 bp (UDO 15), 278 bp and 281 bp (OLEST 7), and 268 bp and 305 bp (OLEST 14), found exclusively in one single accession, most of them in subsp. *laperrinei* (Tables S1 and S2). The unweighted pair group **method** with arithmetic mean (UPGMA) dendrogram (Figure 1) showed the genetic relationships among the 119 genotypes analyzed, based on the set of 12 microsatellite loci used. Genetic similarity ranged from 0 to 1.00, with a value of the cophenetic correlation coefficient r = 0.73 (*p* < 0.001) interpreted as a good fit for clustering analysis. The clustering analysis based on Dice coefficient showed two reallydifferent clusters where the subsp. *laperrinei* was in a single cluster showing a Dice coefficient range between 0 and 0.38. The main cluster included cultivated and putative wild olive genotypes. These genuine olive genotypes did not show a restricted distribution in a single sub-cluster except for WO Kella, Wo Ouled tag, and WO Atounsi but wide genetic variability was however detected among them (Dice coefficient range from 0.48 to 0.7). In the main cluster, six sub-clusters were distinguishable. Two sub-clusters showed a high genetic diversity among olive genotypes even if collected under the same province such in the case of accessions ‘Ballouti’ (Zareza mila) and the accession ‘Messisni ronde’ (Old city mila) 40 km far from each other. Overall, no clear grouping of cultivars in relation to geographical location was observed, except for subsp. *laperrinei* but clustering seemed mostly related to the fruit end-use (Table S3). The majority of genotypes used as table olives and/or dual use were grouped in a distinct sub-cluster. It is worth to note that three putative wild (or feral) olive trees (‘WO Harrouche’, ‘WO Bourghoud 2’, and ‘WO Bourghoud 1’) characterized by large fruit size were included in this cluster.

Even though the Algerian accessions showed high genetic variability, several putative synonymy cases and/or strict relationships were observed among the following combinations of genotypes: ‘Ballouti amzel’/‘Ballouti’, ‘Djbaili’/‘Hamra Agarou’, ‘Blanquette’/Blanquette de Gastu’/‘Rougette’, ‘Balbale Azzaba’/‘Blilti’,’ ‘Bouchouk soummam’/‘Aghenfans’, ‘Aguenanou’/Agrarez’, ‘Bessbassi’/‘Ghreb’/‘Ferdel’, ‘Balbal Bouterdjil’/‘Messisni’, ‘Aeleh’/‘Abani ezzaouia’/‘Abani’, ‘Boukehile’/‘Hamraoui touil’, ‘Hamray azzaba’/‘Zeboudj’.

**Figure 2:**
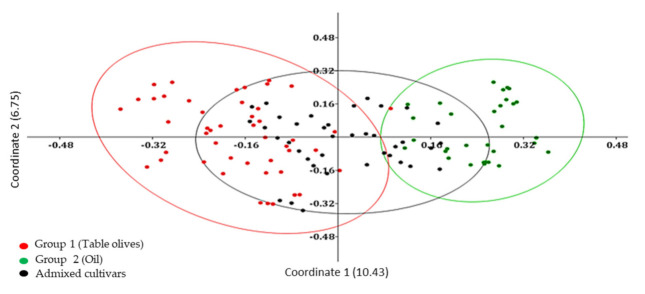
Principal Coordinates Analysis (PCoA) discriminating among 119 Algerian olive accessions based on use-destination.

Genetic relationships among Algerian olive genotypes were highlighted also by using a Principal coordinate analysis (PCoA) that generated a total variation of 17.18% (Figure 2). The first and the second principal coordinates explained 10.43% and 6.75% of genetic variation, respectively. The axes distributed the 119 accessions into main groups following UPGMA clustering and presenting weak relationships. We observed two principal groups, in each main group clusters dispersed according to the type of fruit. The genotypes used as table olives and oil production were grouped into two separated group 1 and group 2, respectively.

### 2.2. Population Structure and Molecular Variance Analysis

**Figure 3:**
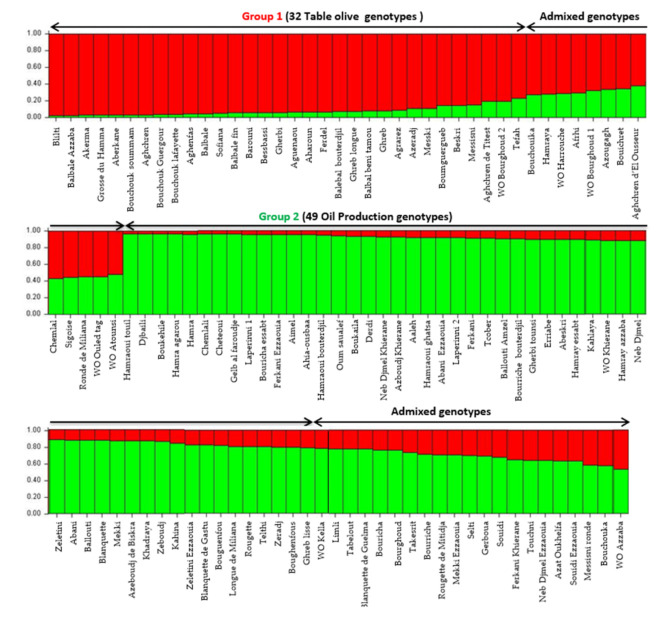
The genetic population structure of 119 Algerian genotypes identified by the STRUCTURE algorithm at K = 2. The red bar includes the first group; the green bar includes the second group; admixed accessions include samples not assigned to a single group.

Population structure was investigated using model-based Bayesian clustering applying STRUCTURE analysis without prior information. Olive tree accessions were grouped in two groups of distinct genetic pools (K = 2) and several admixed accessions (Figure 3). Algerian accessions seemed to be grouped, according to the end use (oil or table) and the size of fruit as previously highlighted by both the UPGMA and PcoA cluster analysis. A threshold of >80% membership q value was used for the designation of group representatives. The first group included 32 genotypes (table olive) and one putative wild accession (WO Bourghoud 2) characterized by a very high fruit size. The second group was composed of 49 genotypes used for oil production, 4 cultivars for double use (‘Guelb al Feroudje’, ‘Tcober’, ‘Abeskri’, ‘Gherbi tounsi’), one wild putative accession (WO Khierane) and two accessions of subsp. *laperrinei*. The other 41 genotypes (33.6%) were assigned to admixed genotypes.

AMOVA was calculated between the two genetic groups according to structure analysis results (Table 2). The observed PhiPT value was highly statistically significant (Fst = 0.062) when a random permutation value of 9999 was used indicating low-moderate genetic differentiation among the sub-populations tested. Most of the genetic variability could be explained by differences within individual referred to a whole population (89%), while 5% and 6% of the total variance was observed within single sub-population and among sub-populations, respectively.

### 2.3. Algerian Accessions Relationships with the Central-Western Olive Germplasm

A cluster analysis aimed to evaluate the occurrence of synonymy cases and highlight relationships among the Central-Western olive germplasm, was carried out on a sample of 461 individuals overall. Interestingly, new strict relationships characterized by very high similarity index were brought to light (Figure S1; Table S4). In this case, the dendrogram could also be divided in two main clusters with subsp. *laperrinei* included in a single group. All the other genotypes were included in the second cluster with several differentiated sub-groups. In general, Tunisian germplasm was found in almost all sub clusters.

In a first sub cluster, a relationship was found between the Sicilian variety ‘Cerasuola’ and the group belonging to the Tunisian variety ‘Zarrazi’ (Dice similarity index: 0.75–0.93) where the accession ‘Sic_45’ corresponded to the authenticated variety ‘Zarrazi’ held in the CREA-OFA collection. In the same sub cluster, the Spanish cultivar ‘Morrut’ clustered with a ‘Chemlali’ group belonging to Tunisian genotypes with a range of similarity beteen 0.7 to 0.82. The accession ‘Sic_22’ was strictly related to the Tunisian accession ‘Rkhami3’ (0.87). Furthermore, the Algerian accessions ‘Ferkani’/’Ferkani Ezzaouia’were really similar to the Tunisian genotypes ‘Chemchali Gafsa’ (0.93).

A second sub cluster included 19 Algerian accessions, the French cultivars’ Picholine’, ‘Oliviere’, ‘Picholine di Languedoc’ and ‘Bouteillan’ and the Spanish varieties ‘Arbequina’, ‘Arbosana’, ‘Sabatera’, Joanenca’, and ‘Perafort’ and 8 Sicilian genotypes, three belonging to known cultivars (‘Pidicuddara’, Patrinostraru’, ‘Zaituna’).

Another very large sub cluster included most of the Spanish varieties with a wide range of variability. Only 3 Algerian accessions were included in this sub cluster. Relationships were found between Sicilian and Spanish olive germplasm, such as between ‘Pizzutella’ and ‘Ojo de Liebre’ (0.76), ‘Manzanilla de Jaen’ (0.86), and the unknown accession ‘Sic_33’ and ‘Dulzal’ (1).

A really interesting sub cluster including 31 Algerian genotypes, 40 Sicilian genotypes, both accessions and known cultivars, and 3 Spanish varieties ‘Manzanillo de Cabra,’ ‘Temprano’ and ‘Gordal Sevillana’. The latter Spanish variety showed strict relationships with the Sicilian varieties ’Pizzo di Corvo’/‘Giarraffa’ (0.87, 0.91) and the Algerian genotypes ‘Balbale Fin’, ‘Blilti’, and ‘Grosse du Hamma’ (0.82–1).

In a very ‘mosaic’ little subcluster we found Spanish, Algerian, Tunisian, and Sicilian germplasm where the Sicilian cultivar ‘Nocellara del Belice’ showed strict association with Tunisian genotypes. The Algerian accession ‘Gelb al Faroudi’ showed very high similarity with the Sicilian accession ‘Sic_2’ (0.94).

Also noteworthy was the substantial genetic uniformity detected between the Sicilian cultivar ‘Sant’agatese’ and the Spanish cultivar ’Cirujal’ (1). The Algerian ‘Messki’ clustered strictly with the Tunisian accession named ‘Meski’ (0.97) and with the Spanish cultivar ‘Fulla de Salze’ (0.85).

A well differentiated sub cluster was found including mostly Algerian accessions, 2 Sicilian cultivars ‘Minuta’ (similar to ‘Vaddara’ and ‘Monaca’), ‘Mandanici’ and ‘Cacazzara’. Only one Spanish cultivar (‘Empeltre’) was found in this group. In this group, other strict relationships were also found among Algerian and Tunisian germplasm: ‘Blanquette’/’Rougette’/’Sayali3’/’Chetoui’/’Zeradj’/’ChaibiOntha’/’Tcober’/’Blanquette de Guelma’/’Haouaria’ and ‘Gerboua’ accessions with a variable range of similarity between 0.73 and 1.

Private alleles in the Algerian germplasm were further confirmed by comparison of molecular profiles with the accessions from Tunisia, Sicily, Spain and France (Table S2) except in one case. Interestingly, the 167 bp allele at the locus GAPU 71b previously found exclusively in subsp. *laperrinei* was also detected in a few Spanish cultivars, such as the ancient variety ‘Farga’.

### 2.4. Population Structure of the Central-Western Olive Germplasm and Molecular Variance

The genetic population structure analysis was conducted on the panel of olive tree accessions excluding putative synonym cases. The Bayesian clustering model implemented in STRUCTURE software indicated K = 2 as the best number of sub-populations (Figure S2) confirming substantially the dendrogram results. Two main groups were in fact highlighted with the most of Algerian samples included in the first group while the most of Spanish varieties were included in the second one. Admixed genotypes were assigned when membership value was less than 80% [2,18] and in according to this criterion, a rather high number of admixed genotypes were individuated. These results reflect the framework of genetic pattern of diversification of cultivated olive in the Mediterranean basin already previously described [2,3,5] except for the 2 accessions of subsp. laperrinei which clustered in the same group of Algerian accessions. This latter group included in fact 48 accessions among Algerian cultivars, wild olives, and subsp. laperrinei. In the same group were included also 3 French cultivars (‘Oliviere’, ‘Zinzala’, and ‘Picholine du Languedoc’), 17 known Sicilian varieties, 13 unknown Sicilian accessions, 12 Spanish cultivars among which the cultivars ‘Arbequina’, ‘Empeltre’, and ‘Farga’. Furthermore 17 Tunisian genotypes were included in this group. The second group comprised 57 Spanish varieties and the only Moroccan variety included in the dataset while a much more reduced number of genotypes from Central Mediterranean basin were found in this group: 3 Sicilian varieties (‘Giarraffa’, ‘Moresca’, and ‘Vallanella’), 3 Sicilian unknown accessions, and 3 Algerian varieties. No Tunisian genotypes belonged to this group. Admixed genotypes included most of Sicilian germplasm (49 accessions) both known cultivars (21) and unknown genotypes (28). Overall, 25 accessions clustered next to the ‘Algerian group’ showing a membership threshold slightly lower than 80% while 18 accessions shared most of their gene pool with the ‘Spanish group’. However, it is worth to note a high admixture level was found for all the genotypes tested with different geographic provenience sharing most of their alleles with the ‘Algerian group’.

Molecular variance analysis (AMOVA) was conducted on the two differentiated groups highlighted by STRUCTURE using the same previous described parameters. The observed PhiPT value was highly statistically significant (Fst = 0.274) indicating high differentiation level between the two groups explaining 27% total variance. However, genetic variation within population explained most of the total variance (Table 3).

### 2.5. Diversification Process: Evidence of Bottleneck, Parentage Analysis, and Demographic Modelling

Evidence for a bottleneck was evaluated using the genetic differentiated groups individuated by STRUCTURE analysis and here treated as two divergent populations. Three different tests were conducted to detect significant deviations from mutation-drift equilibrium but no significant excess of heterozygosity was detected under one-tailed Wilcoxon rank test. As described above, the two genetic groups did not reflect completely geographic localization of the genotypes here analyzed. In order to evaluate a putative ‘geographic effect’ and infer new hypothesis about diversification process of cultivated olive in the Mediterranean basin, we tried to evaluate bottleneck on dataset including only Spanish and Algerian varieties differentiated by Structure analysis. No evidence of signature for a bottleneck was found in Algerian population while a significant deviation from mutation-drift equilibrium was observed for the sample of 57 Spanish varieties included in the dataset (*p* = 0.027) confirming results obtained by Diez et al. [2]. Parentage analysis conducted on the whole dataset from the Central-Western Mediterranean basin (without putative synonyms) as candidate parents (324) revealed critical LOD scores of 17.77 and 13.08 for the parent pair analysis with unknown sexes for strict (95%) and relaxed (80%) confidence levels, respectively. The main putative pairs of parents, without mismatch between loci are shown in Table S5. Several Algerian accessions were included in offspring combinations with genotypes from all the geographic proveniences. The Algerian accessions ‘Bouchouk Guergour’, ‘Mekki Ezzaouia,’ ‘Bouchouika’, ‘Sofiana’, ‘Aghenfas’, were more frequently detected in offspring combinations. Spanish germplasm was also involved in several offspring combinations with genotypes of different provenience, in particular the varieties ‘Limoncillo’, ‘Redondilla de Logroño’, ‘Escarabajuelo de Atarfe’, ‘Escarabajuelo de Posadas’, ‘Manzanilla de Abla’, ‘Habichuelero de Grazalema’, ‘Alfafara’. More in general, higher number of ‘Spanish’ offspring combinations were detected, confirming the bottleneck event as favoring the allele fixation in the Spanish population.

Sicilian varieties such as ‘Giarraffa’, ‘Pizzutella’, and ‘Sant’agatese’ were also included in several offspring combinations. The ‘Giarraffa’ being really genetically similar to the Spanish cultivar ‘Gordal Sevillana’ was included in offspring combinations with several Spanish cultivars and this is an aspect that can mislead the results on the genetic relationships between olive varieties, highlighting the importance to identify potential cases of synonymy for a correct interpretation.

However, also ‘Pizzutella’ matched with several Spanish and Algerian varieties. Strict relationships with Spanish germplasm were found also for ‘Sant’agatese’ belonging to the ‘Algerian group’ from the structure analysis. Interestingly the old Spanish variety ‘Farga’ was included in this offspring combination. Several unknown Sicilian accessions showed know Sicilian cultivars as putative parentals, but in a few cases also varieties from other proveniences confirming Sicily as a region really rich in olive trees biodiversity due to its geographical position in the center of the Mediterranean which has favored the passage of many civilizations. The French varieties here considered showed offspring combinations with different provenience genotypes.

**Figure 4:**
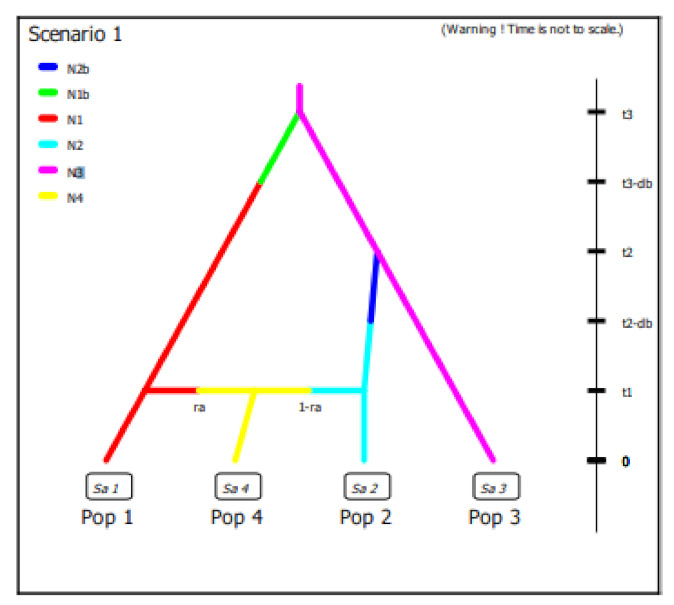
The best supported scenario by the observed data calculated using ABC. In this scenario an independent origin of Pop1 was hypothesized (Algeria sample) and Pop 2 (Spanish sample) and one recent admixture event (Pop 4) with 70% rate of admixture (ra) from Pop1 Historical parameters: t3: 300; t2: 280; db: 50; t1: 88; N1: 47; N2: 57; N3: 2; N4: 74; ra: 0.7, N1b: 40, N2b: 50.

In this work we did not have different samples of wild olive accessions available to build a scenario able to elucidate domestication process but we decided to evaluate the best fitting demographic model with observed data using Approximate Bayesian Computation (ABC) in order to clarify the diversification process of cultivated olive around the Central-Western Mediterranean basin. Considering the results obtained on the diversity and genetic structure of the Algerian population per se and compared with the germplasm of the Central-Western Mediterranean basin as well as the hypothesis still open on domestication centers, we defined 4 historical scenarios using subsp. *laperrinei* as common ancestral lineage although a low number of samples were available (Figure S3). To simplify completely the model, we used a dataset with differentiated populations by STRUCTURE analysis coming from Algeria and Spain exclusively and their admixed accessions. In the scenarios definition we considered both divergence and admixture events (Figure S3). Since, in general, every population divergence event is followed by a bottleneck, the ABC asks to include this kind of event. Because of long generation time of olive tree, we assumed a generation time of 20 years as indicated by Diez et al. [2]. All the parameters of the historical models were described in Figure S3. The posterior probabilities of scenario were computed by local linear regression using the 10 subsets of simulated data closest to observed data. The results showed for all the 10 subsets of data a 99% confidence interval for the scenario 1 indicating this scenario was the best supported by observed data (Figure 4). We also evaluated the posterior error rate given as a proportion of wrongly identified scenarios over the 1000 test data sets for both the direct and the logistic approaches. The global prior error rate for scenario 1 was 0.38. We also evaluated the prior error rate for a specific scenario that allows to estimate two type of errors: type I error which is the probability to reject the true scenario and type II error that indicates the probability to accept a wrong scenario. The probabilities to commit both the I and II error type were very low, ranging from 1.7 to 2.6% and from 4.9 to 5.5%, respectively, considering each specific scenario.

## 3. Discussion

The first purpose of this work was to improve knowledge on the genetic diversity and population structure of Algerian olive germplasm. For this aim, a set of 12 SSR markers was used for genotyping of 109 cultivars, 8 putative wild (or feral) accessions and 2 individuals belonging to subsp. *laperrinei*. The average number of alleles here observed was higher than those obtained in previous works [15,19]. The expected and observed heterozygosity averages in our study were higher [1,12] or comparable [10,20] with those observed in previous works aimed to Algerian olive germplasm genotyping. These results confirmed the high genetic variability of Algerian olive germplasm. Based on the Polymorphism Index Content (PIC) and Shannon’s information index (I) values, we could classify the used primers according to their effectiveness. The most suitable loci for the genetic characterization for the analyzed genotypes were DCA 18, DCA 3, and DCA 8, which showed polymorphism equal to or higher than 80% and Shannon’s information index more than 2, results in accordance with previous studies [11,21]. Among the 127 alleles found in genotyping of the Algerian accessions, a consistent number of private alleles was detected indicating that a putative isolation process might have occurred. For instance, several private alleles were detected in the subsp. *laperrinei*, one of the first subsp. of the *Olea* complex that has differentiated following successive phases of aridification of the Saharan region since the Late Miocene [3]. Cluster analysis in our study confirmed an early differentiation, in line with previous studies which clearly indicated phenotypic and genetic divergence between subsp. *laperrinei* and cultivated olive (subsp. *europaea* var. bot. *europaea*), while maintaining the interfertility [4,8,22]. However, cluster, PCoA and genetic structure population analysis did not detect a grouping related to geographical location for cultivated and putative wild (or feral) olive trees, but the clustering seemed to be related mostly to the fruit end-use, in accordance with the previous results obtained by AFLP, RAPD, and SNP markers [23,24,25]. AMOVA analysis indicated further a weak differentiation structure detected in our set of analyzed samples. A low Fst value was expected due to the lack of geographical discrimination observed and large distribution of genetic variability detected between cultivated and putative wild (or feral) olives.

Our results highlighted several groups of synonyms and homonymies, probably due the widest sample ever collected before for Algerian germplasm characterization. In this study, 46 accessions (including two accessions of subps. *laperrinei*) from ITAFV official Algerian collections were analyzed giving new molecular information for further 10 cultivars (‘Aghchren’, ‘Azeboudj de Biskra’, ‘Azougagh’, ‘Balbal Beni tamou’, ’Blanquette’, ‘Boumguergueb’, ‘Gerboua’, ‘Rougette’, ‘Zeboudj’, ‘Zeletini’). In Algeria, the nomenclature of olive cultivars occurs in different languages (Arabic, Berbere) and mainly refers to several traits such as the color (Aberkane, Suidi, and Kahlaya for black olives; Azougagh, Hamra, and Hamray for red olives), surface and size of leaves (Bouricha, Bourriche for cultivars with large size leaves), the ripening period (Chetoui in winter and Tcober in October) and their origin (Zeboudj and Azeboudj for wild olive). Because of the long history of cultivation and absence of a common criteria for cultivar classification, these indigenous names may have contributed to the confusion in the varieties cataloguing. Cluster analysis highlighted putative synonymies such as the case of the cultivars ‘Aaleh’ (also named ‘Aeleh’ or ‘Aeleth’), ‘Abani’ and the accession ‘Abani Ezzaouia.’ This result was confirmed also by a previous work [20]. The ‘Aaleh’ and ‘Abani’ cultivars are currently held in the ITAFV collection in Bejaia. However, our results lead us to speculate the ‘Aaleh’ variety held in ITAFV could be not authentic, because the ‘Aeleh’ variety was in the past described as a plant originally from Bejaia, with small-rounded olives grouped at the base of the twigs [26] whereas the ‘Aaleh’ variety from ITAFV collection was collected in Chechar Khenchela district and morphologically described with elongated fruits, really similar to ‘Abani’ cultivar [9]. Lastly, within an internal survey conducted with local farmers in Khenchela, the ‘Aaleh’ name was never mentioned (Personal communication). The inter varietal discrimination among accessions from ITAFV of Bejaia and their molecular profiles obtained in this study only partially agree with those of previous works [1,10,11,12,20], raising the doubt that there is still some confusion in the management of Algerian germplasm and highlighting that varietal authentication processes are still very necessary.

The dendrogram showed near-synonyms cases whose allelic profiles differed at ≤2 loci. Previous studies explained these slight differences probably due to spontaneous somatic mutations reported as a frequent event in long-lived olive trees propagated by cloning [27,28,29]. In addition, we observed twelve cases of homonymies involving the following cultivars: ‘Ferkani’, ‘Neb Djmel’, ‘Mekki’, ‘Souidi’, ‘Zeletini’, ‘Bouricha’, ‘Hamray’, ‘Balbale’, ‘Bourriche’, ‘Hamra’, and ‘Hamraoui’ collected from different locations (ITAFV collections, Skikda, Khenchela, and Mila). More than one accession shared the same basic name but the dendogram clearly showed their genetic diversity. These cases of homonymy in Algerian germplasm included most cultivars maintained in situ and never characterized before, except a few minor accessions previously reported [10,12,30].

Ancient fossil studies in North Africa [31,32] have mentioned that the wild olive had existed in many Saharan sites much earlier than the 12th millennium BC [32]. Therefore, a domestication event is likely to have occurred in the Auras region with further spread of the olive tree grown in the rest of Algeria. We found a close relationship between different varieties from the Auras region which includes the provinces of Batna, Biskra, Tebessa, and Khenchela. These cultivars belong to two subgroups in the dendrogram and the same STRUCTURE group, showing the same end-use. In the Auras region, olive groves have remained confined along the valleys and mountainous areas and this region is witnessing a significant decline in genetic resources due to environmental changes. Varieties from this region have adapted to difficult environmental factors, such as high temperatures, and should be considered an attractive genetic resource for adaptive traits. Accessions from Auras region were clustered in a differentiated group (i.e., ‘Erriabe’, ‘Abeskri’, ‘Azeboudj Khierane’, etc.) showing similar end-use. Relationships were due to the geographical isolation which gave rise to admixed genotypes only in a few cases. Phylogenetic analysis here conducted were consistent with the previous fossil studies [31,32] led us to hypothesize an ancient origin for this group of varieties.

In this study, no clear distinction was observed between cultivated and putative wild olives in agreement with other previous studies [33,34]. New genuine olive accessions were here included in the Algerian olive germplasm biodiversity.

Likewise, to provide new insights about the olive diversification history and other evolutionary events in the Central-Western Mediterranean basin, Algerian germplasm was compared with other cultivars and accessions from the Central-Western Mediterranean basin. The dendrogram and structure analysis showed a differentiation of the Central-Western Mediterranean olive germplasm in two groups: the group of Spanish varieties which exhibited a characteristic bottleneck and the ‘Algerian’ germplasm which includes the greater part of the Mediterranean germplasm analyzed here.

High admixture rate was observed especially for Sicilian germplasm sharing most of ‘Algerian’ gene pool. These results confirmed the pattern of diversification of cultivated olive germplasm and bottleneck evidence previously described [2]. The ‘Algerian’ genepool of this study could be referred to group Q2 while the ‘Spanish’ germplasm could be referred to Q1 [2,5].

In this case, cluster analysis conducted among Algerian olive germplasm and other cultivars and accessions from the Central-Western Mediterranean basin also highlighted a clear differentiation of susp. *laperrinei.* However, structure analysis included the accessions belonging to this subsp. in the same group of the ‘Algerian’ germplasm. Haddad et al. [1] found the highest proportion of Algerian varieties shared the same haplotype with the majority of Mediterranean cultivars (clorotype E1) and the Laperrine’s olive, speculating about a local domestication process from oleasters and this subspecies. Besnard et al. [2] found a plastidic lineage shared among cultivated and Laperrine’s olive not present in oleaster. Most of the private alleles previously found in the Algerian germplasm were not found in the rest of Mediterranean germplasm while a few of them and some rare alleles were found almost exclusively in Mediterranean germplasm belonging to ‘Algerian group’ and some admixed genotypes sharing most of ‘Algerian’ alleles. The two ‘Laperrinei’ accessions carry mutations that were fixed in the population and maintained as result of ancient isolation, resulting in private alleles. However, in one case, the allele 167 bp at the locus DCA18 was detected in 6 Spanish varieties sharing the ‘Algerian’ gene pool as in the very old cultivar ‘Farga’. The presence of the 167 bp allele found exclusively in these Spanish varieties led us to consider a different temporal and spatial origin of Spanish germplasm.

Cluster analysis highlighted several cases of putative synonymy leading us to speculate about the path of diffusion of important traditional cultivars such as ‘Giarraffa’ and ‘Gordal Sevillana’ from Sicily and Spain, respectively, by vegetative propagation. In this study we found out a new strict relationship of these cultivars with Algerian genotypes such as ‘Balbale Fin’, ‘Blilti’, and ‘Grosse du Hamma’ belonging to ‘Spanish’ groups as showed by STRUCTURE analysis. The parentage analysis included ‘Giarraffa’ in several offspring combinations without mismatches with a few Sicilian, Tunisian and Spanish accessions/cultivars. Diez et al. [2] indicated the old cultivar ‘Gordal Sevillana’ as putative parental line of modern Spanish cultivars. Afterwards, more recent admixture events occurred giving rise to admixed cultivars such as ‘Moresca’, ‘Lumiaru’, ‘Ogliarola Messinese’, and other unknown Sicilian accessions here analyzed. In Algeria we found the ‘Blilti’ genotype as putative parental line of the ‘WO Harrouche’. The Algerian variety ‘Sofiana’ with strict relationships with ‘Gordal Sevillana’/’Giarraffa’ could be a putative parental line of admixed varieties such as ‘Lumiaru’ and ‘Bouchouika’. We hypothesized that the Spanish variety ‘Gordal Sevillana’ was spread by vegetative propagation along the southern coasts of the Mediterranean, until reaching Algeria and Sicily.

Most of synonymy cases for Algerian germplasm were found with Tunisian genotypes. A relatively low number of Tunisian alleles were included in the analysis but it is reasonable to assume that an active exchange of vegetatively propagated material probably took place due to the closeness of these two countries.

The demographic analysis confirmed our hypothesis: the best supported model was the scenario where we hypothesized an independent origin of Algerian and Spanish populations from each other and where recently they gave origin by a ‘mosaic’ (admixture) population. Furthermore, in this model we set up a more recent origin for the Spanish population than the Algerian germplasm. Diez et al. [2] observed that the Spanish group Q1 derived from a more recent admixture event between wild east (WE) and wild west (WW) oleasters while Q2 seemed to have diversified earlier and originated from Q3 and WW. Although, we had neither wild olive population nor cultivated olive accessions from East Mediterranean area available for inferring historical and genetic evolution of cultivated olive, our results seem to confirm an independent evolution of Algerian and Spanish populations. The admixed population here found by structure analysis showed a recent origin consistent with the ‘mosaic’ population described by Diez et al. [2]. Admixture events probably occurred by historical migrations during the existence of ancient Maghreb. In fact, our study included cultivars and accessions from Algeria, Tunisia, Morocco, Sicilia, Spain (formerly defined El-Andalus), and France, countries that historically concurred to form the ancient Maghreb. Furthermore, we hypothesize that the spreading of ‘Gordal Sevillana’ towards Sicily and Algeria, giving rise to local admixed varieties and accessions, could be due to the more recent Islamic invasion.

On the other hand, the STRUCTURE analysis included some Spanish varieties within the ‘Algerian’ group such as the ancient ‘Farga’ variety and the ‘Cirujal’ cultivar genetically very similar to the Sicilian ‘Sant’Agatese’ cultivar. Diez et al. [2] found the ‘Cirujal’ cultivar in the Q3 group, including the eastern cultivars and from which the Q2 group originated. Our results seem to confirm an early diversification of the ‘Algerian’ gene pool probably widely spread by the Phoenicians along the African and Sicilian coasts starting from the Levant.

The large diffusion of the ‘Algerian’ gene pool throughout the Central Mediterranean countries here found corroborates the hypothesis a very early diversification process might have occurred.

## 4. Materials and Methods

### 4.1. Plant Material

**Figure 5:**
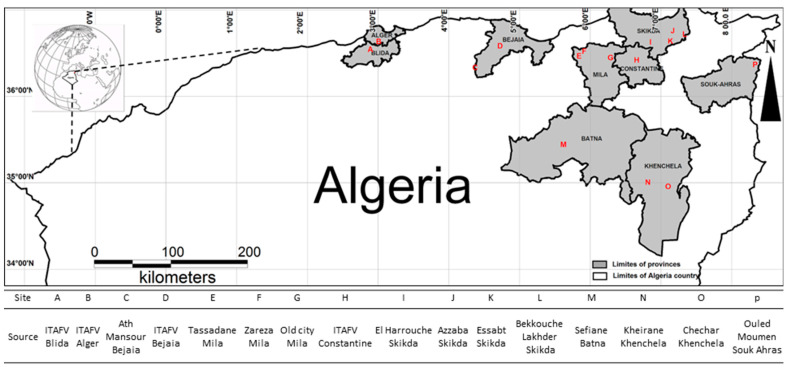
Map of geographical distribution of collected Algerian samples in this study.

In this work, 119 Algerian olive tree accessions including 109 cultivated varieties, 8 putative wild (or feral) olive trees (WO), and 2 accessions of *Olea europaea* subsp. *laperrinei* were collected and georeferenced (Table S3; Figure 5). Among the cultivated olive varieties, 44 accessions were obtained from the National Germplasm collections held at the Institut de l’Arboricolture Fruitiére et de la Vigne (ITAFV) located in several cities: 35 varieties were collected from Béjaia Tekerietz, 8 varieties from Blida (Beni Tamou), 1 variety from Constantine (El Hama), while 2 accessions belonging to the sub-sp. *laperrinei* were collected from Alger (Tassala elmerdja). The other 76 local accessions (cultivated and WO accessions) were collected in situ both in natural sites and private orchards. The sampling was conducted using different geographical distribution as criteria in the eastern Algeria: 8 accessions from Batna, 1 from Bejaia, 20 accessions, and 1 WO tree from Khenchela, 21 accessions and 5 WO trees from Tassadane Mila, 12 accessions and 2 WO trees from El harrouche Skikda, and 6 accessions from Souk Ahras. A population of 52 Sicilian unknown accessions and a set of 51 reference Sicilian varieties were included in the analysis (Table S2) for the comparison between Algerian samples and the olive germplasm from Central-Western Mediterranean Basin. The Sicilian varieties were collected at the Council for Agricultural Research and Economics-Research Center for Olive, Fruit and Citrus Crops (CREA-OFA) varietal collection field located in Mirto, on the Ionian coast of the province of Cosenza, Italy. The unknown accessions were collected under different geographical locations in Sicily region.

### 4.2. DNA Extraction

Genomic DNA was extracted from 50 mg finely ground powder of young leaves using a commercial kit (Plant DNA Mini Kit, Qiagen, Hilden, Germany). DNA quality and concentration were checked with a NanoDrop 2000 spectrophotometer (Thermo Scientific, Waltham, MA, USA), diluted to 20 ng/μL and stored at −20 °C until used.

### 4.3. Microsatellite Analysis

The following twelve labelled microsatellite markers (SSR) were selected and used for molecular analysis in 4 multiplex combinations: DCA3-6FAM, DCA5-VIC, DCA8-VIC, DCA11-PET, DCA18-6FAM [35], EMO90-NED [13], GAPU71b-6FAM [36], OLEST7-PET, OLEST14-VIC, OLEST23-PET [37], and UDO12-NED, UDO15-NED [27] (Table S6). These microsatellite markers were chosen according to their level of polymorphism degree in Algerian olive trees [1,10,12,30], reproducibility, ease of reading and suitability for multiplex amplification strategy [21]. Amplification by PCR for all the multiplex combinations was carried out in a total volume of 15 μL, containing 10 ng of genomic DNA, 10× PCR buffer, 2 mM MgCl_2_, 2.5 mM dNTPs, 10 μM of forward and reverse primers, and 5 U/μL Taq polymerase, using a thermal cycler (GeneAmp PCR System 9700 Applied Biosystems Inc., Foster City, CA, USA). The PCR thermal profile was programmed as follows: a first step at 94 °C for 5 min, 30 cycles at 94 °C for 30 s, 55 °C for 30 s, and 72 °C for 40 s. The last step included 7 min of incubation at 72 °C. We also included two reference varieties (Leccino and Frantoio) in PCR amplification to check experimental conditions (data not shown). The GeneScan 500LIZ (Life Technologies, Carlsbad, CA, USA) was used as internal standard and the SSR migration was carried out using the Applied Biosystems SeqStudio Genetic Analyzer (Thermo Fisher Scientific Inc., Waltham, MA, USA).

### 4.4. Data Analysis

The amplified peaks were analyzed using the Gene Mapper v.6.0 software. In order to get a more complete framework of olive relationships in the Central-Western Mediterranean basin, molecular data results from Algerian and Sicilian samples were compared with 154, 74, 10, and 1 molecular profiles of cultivars from Spain, Tunisia, France, and Morocco, respectively, using common markers found in literature [18,38,39,40] in a range from 4 to 8 loci, and included in our internal database of the Council for Agricultural Research and Economics-Research Center for Olive, Fruit, and Citrus Crops (CREA-OFA). Before comparison, all SSR markers were subjected to a “binning” following the methodology described by Ben Mohamed et al. [39]. For each locus SSR, the alleles detected were scored as present (1) or absent (0), then a similarity matrix using Dice’s coefficient [41] was first obtained and used to determine the cluster analysis based on the unweighted pair group method with arithmetic mean (UPGMA). A dendrogram and cophenetic correlations were obtained using Past software v.2.12.

To avoid considering variants of the same accession in the data analyses but also to avoid including families of very closely related individuals that can bias Bayesian clustering analyses [42], we decided to keep the molecular profile of known reference cultivars or in the case of accessions we chose randomly only one tree for such closely related accessions. Then, all genetic diversity and structure population analysis were carried out on a sample of individuals considered to have a unique profile.

The number of alleles per locus (A), their frequency (Fa), observed (Ho), and expected heterozygosity (He), polymorphism information content (PIC), were calculated using Cervus v.3.0.7 [43]. The Wright’s inbreeding coefficient (Fis), the fixation index (Fst), the number of null alleles (F null), and the deviation from the Hardy–Weinberg equilibrium (HW) corrected using the Bonferroni method for each microsatellite locus and gene flow (Nm) estimates were calculated using PopGene software v.1.32 [44]. Shannon’s information index (I) and the Analysis of Molecular Variance (AMOVA) were calculated using GenAlEx 6.5 using 9999 random permutations [45]. Analysis of the genetic structure populations was performed using a classification method based on a Bayesian model implemented in STRUCTURE v.2.3.4. software [46]. The admixture model with correlated allele frequency and a burn-in length of 100,000 followed by 100,000 runs at each K with three iterations for every K were used, with K ranging from 1 to 12. Then, the Structure Harvester web version 0.6.93 [47] was used to select the optimal K, using ΔK, according to Evanno et al. [48].

A test for evaluation of signature of genetic bottleneck was conducted using Bottleneck v.1.2.02, under the two-phase mutation model (T.P.M.) setting up 70% stepwise mutations and a variance among multiple steps of 12.

Relatedness between individuals was evaluated using a parentage analysis computed using Cervus v.3.0.7 software. An approach based on the logarithm of the odds (LOD) score significance was adopted and the following parameters were run: (i) number of offspring: 200,000; (ii) number of candidate parents: 324; (iii) proportion of candidate parents sampled: 0.7; (iv) proportion of loci typed: 0.8218. Default values were adopted for the parameters “proportion of loci mistyped” and “error rate in likelihood calculations”. The relaxed and strict confidence levels were set up to 80% and to 95%, respectively.

An Approximate Bayesian Computation (ABC) with DIYABC software v 2. [49] was used to compare possible scenarios for the same data file through the computation of the posterior probabilities of each scenario. For the ABC simulations of scenarios, a generalized stepwise model (GSM) is adopted where historical and mutational parameters are used to build each scenario. Mutational parameters refer to the mean mutation rate (µ) and the mean parameter (P) of the geometric distribution with the aim to model the length of mutation events. In this work, we adopted the same mutational parameters and summary statistics indicated by Diez et al. [2], maintaining the allele range state for each locus as a default range (40) for all the SSR loci included in the model.

## 5. Conclusions

In this work we characterized new genetic resources from Algeria which enlarges the olive gene pool. Climate change severely affects the Mediterranean olive growing and new adaptative traits are needed to afford the challenge. A further effort will be needed to evaluate the Algerian germplasm as potentially source of genes encoding adaptative traits for facing olive growing new challenges. The comparison among Algerian olive germplasm and other cultivars and accessions from the Central-Western Mediterranean basin, highlighted new several cases and close relationships among accessions raising a new insight about the importance of vegetative propagation in the olive tree diffusion process along the Mediterranean basin. Demographic analysis corroborated the previous hypothesis of an early differentiation of olive germplasm from Central Mediterranean basin and recent admixture events occurrence confirming the high genetic variability present in the Central Mediterranean area, particularly in the Sicily region. These studies are crucial steps to address the retrieval of genetic resources and begin new pre-breeding activities.

## Figures and Tables

**Table 1:** Diversity parameters detected in the Algerian sample of 119 accessions for each of 12 Simple Sequence Repeats (SSR) marker used: size range, number of detected alleles (No), number of effective alleles (Ne), observed (H_O_) and (He) expected heterozygosity, significance of (HW) test, frequency of null alleles F (null), Inbreeding coefficient (Fis), polymorphic information content (PIC), Shannon’s information index (I). *** High significance; ** significance, NS: not significan, ND: not determined.

Locus	Size Ranges (bp)	No	Ne	Ho	He	HW	F(Null)	Fis	PIC	I
DCA18	167–189	12	8.42	0.95	0.89	ND	−0.036	−0.0786	0.869	2.23
DCA03	228–257	14	6.53	0.86	0.85	NS	−0.007	0.0069	0.828	2.04
DCA08	119–159	16	6.02	0.86	0.84	NS	−0.018	−0.0568	0.812	2.05
OLEST14	268–308	13	5.33	0.84	0.82	NS	−0.019	−0.0146	0.789	1.90
OLEST7	260–281	8	1.72	0.82	0.80	NS	−0.014	−0.0138	0.772	1.72
GAPU71B	121–147	7	4.68	0.86	0.79	NS	−0.045	−0.0953	0.754	1.64
DCA11	130–163	12	1.70	0.48	0.76	***	0.224	0.347	0.719	1.70
OLEST23	203–222	6	1.46	0.89	0.76	**	−0.087	−0.2026	0.711	1.46
UDO15	100–123	10	3.54	0.28	0.72	***	0.454	0.6151	0.678	1.55
DCA05	192–222	14	3.25	0.73	0.69	NS	−0.031	−0.0948	0.670	1.71
UDO12	150–166	8	2.98	0.44	0.67	***	0.209	0.319	0.627	1.37
EMO90	186–202	7	3.09	0.66	0.67	NS	−0.014	0.0008	0.621	1.37
Mean		10.6	4.06	0.72	0.77				0.74	1.73

**Table 2:** Analysis of molecular variance (AMOVA) of 119 Algerian olive accessions.

Source of Variance	DF	SS	MS	Variance Components	F-Statistics	Total Variation	*p*
Among Populations (Fst)	2	57.087	28.543	0.298	0.062	6%	<0.001
Within Population (Fis)	119	560.708	4.712	0.235	0.052	5%	<0.001
Within Individual (Fit)	119	517.50	4.242	4.242	0.112	89%	<0.001

**Table 3:** Analysis of molecular variance (AMOVA) of the Central-Western Olive Germplasm.

Source of Variance	DF	SS	MS	Variance Components	Total Variation	*p*
Among Populations (Fst)	1	261.46	261.46	3.15	27%	0.000
Within Populations (Fis)	164	1368.43	8.34	8.34	73%	0.000

## Supplementary Materials

The following are available online at https://www.mdpi.com/article/10.3390/plants10040678/s1, Figure S1. Dendogram of the Central-Western Mediterranean accessions based on the UPGMA methodology; Figure S2. Structure analysis of the Central-Western Mediterranean olive accessions; Figure S3. The 4 scenarios compared using approximative Bayesian computation; Table S1. Size and frequency of alleles for the 12 SSR loci detected in the 119 olive accessions. In bold are shown private alleles; Table S2. Molecular profiles of the Central-Western Mediterranean olive accessions; Table S3. List of olive accessions collected, geographical origin, georeferentiation and use destination; Table S4. Dice similarity indexes among the 461 olive accessions from Central-Western Mediterranean basin; Table S5. Parentage analysis among Central-Western Mediterranean accessions; Table S6. Primers list, repeat motives, annealing temperature (Ta) and size range for the 12 SSR used for the genotyping of Algerian cultivars.
