# Supplementary material for: Algerian Olive Germplasm and Its Relationships with the Central-Western Mediterranean Varieties Contributes to Clarify Cultivated Olive Diversification
Source: Plants (Basel). 2021 Apr 1;10(4):678. doi: 10.3390/plants10040678 (PMC8066573; doi:10.3390/plants10040678)
Supplement: Supplementary file 1 [file plants-10-00678-s001.zip › SupplementaryMaterial/FIGURE S3.pptx]

## Slide 1
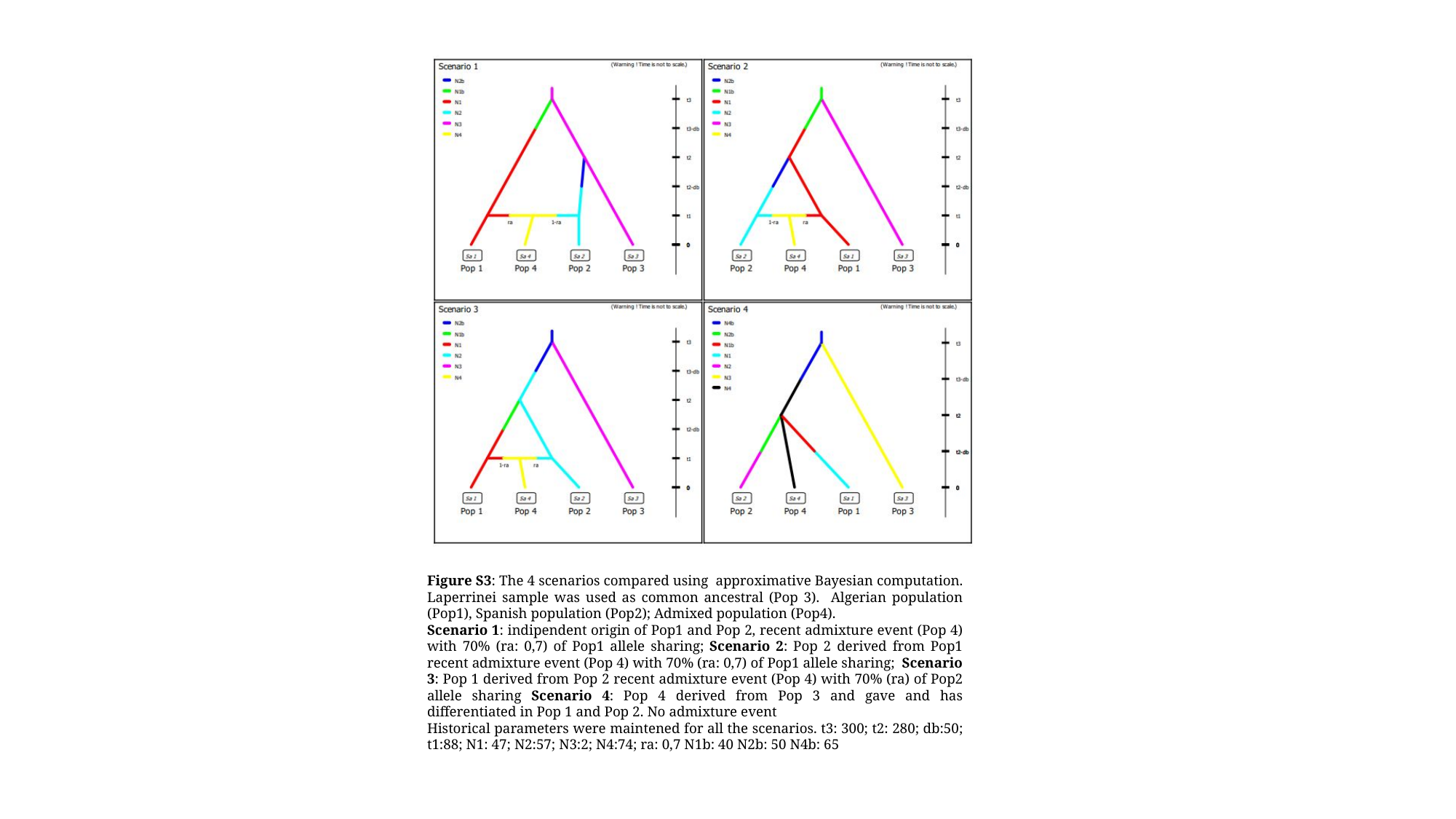

Figure S3: The 4 scenarios compared using approximative Bayesian computation. Laperrinei sample was used as common ancestral (Pop 3). Algerian population (Pop1), Spanish population (Pop2); Admixed population (Pop4).
Scenario 1: indipendent origin of Pop1 and Pop 2, recent admixture event (Pop 4) with 70% (ra: 0,7) of Pop1 allele sharing; Scenario 2: Pop 2 derived from Pop1 recent admixture event (Pop 4) with 70% (ra: 0,7) of Pop1 allele sharing; Scenario 3: Pop 1 derived from Pop 2 recent admixture event (Pop 4) with 70% (ra) of Pop2 allele sharing Scenario 4: Pop 4 derived from Pop 3 and gave and has differentiated in Pop 1 and Pop 2. No admixture event
Historical parameters were maintened for all the scenarios. t3: 300; t2: 280; db:50; t1:88; N1: 47; N2:57; N3:2; N4:74; ra: 0,7 N1b: 40 N2b: 50 N4b: 65
