# Supplementary material for: Algerian Olive Germplasm and Its Relationships with the Central-Western Mediterranean Varieties Contributes to Clarify Cultivated Olive Diversification
Source: Plants (Basel). 2021 Apr 1;10(4):678. doi: 10.3390/plants10040678 (PMC8066573; doi:10.3390/plants10040678)
Supplement: Supplementary file 1 [file plants-10-00678-s001.zip › SupplementaryMaterial/FIGURES1.pptx]

## Slide 1
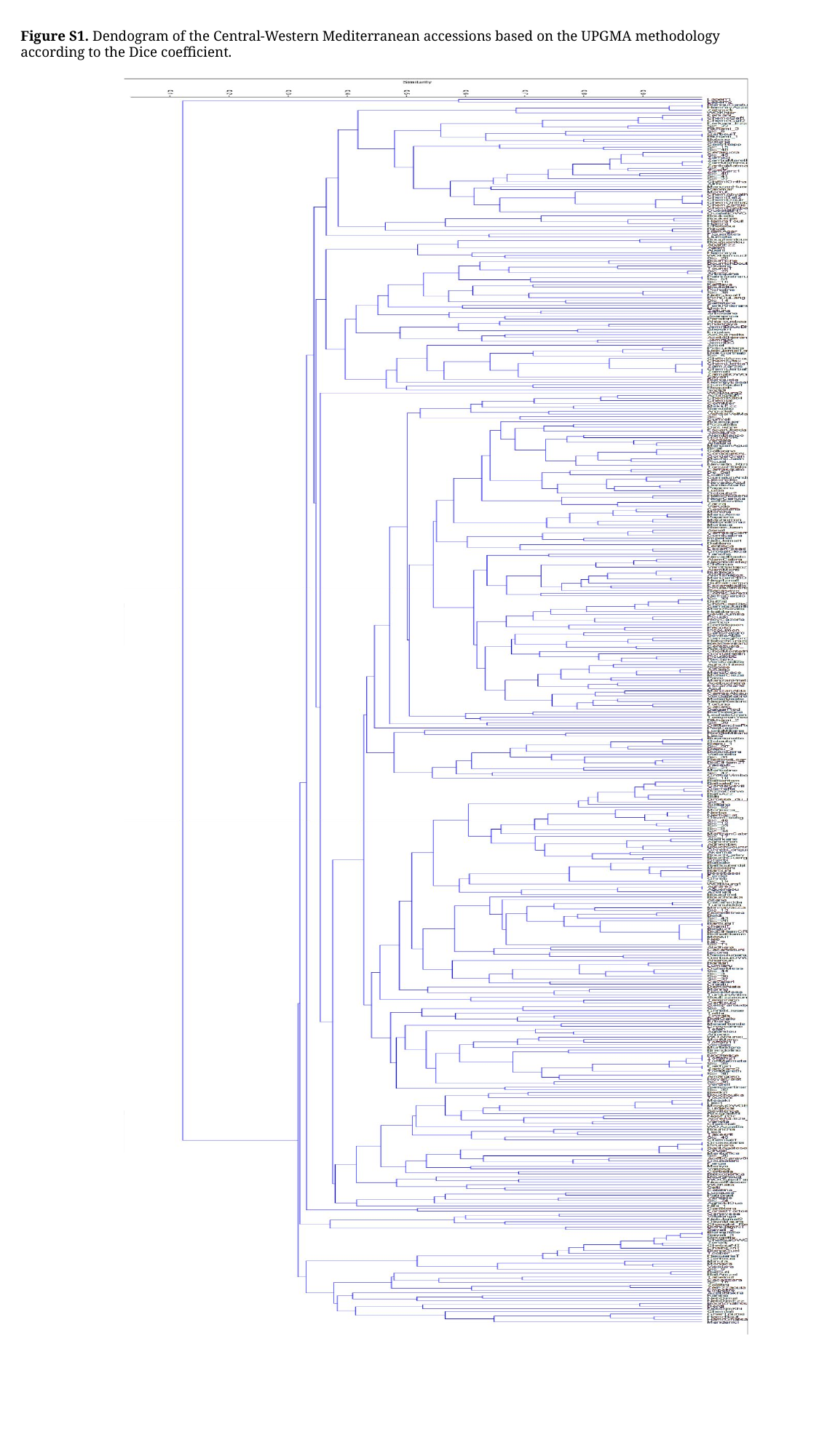

Figure S1. Dendogram of the Central-Western Mediterranean accessions based on the UPGMA methodology
according to the Dice coefficient.
