# Supplementary material for: Algerian Olive Germplasm and Its Relationships with the Central-Western Mediterranean Varieties Contributes to Clarify Cultivated Olive Diversification
Source: Plants (Basel). 2021 Apr 1;10(4):678. doi: 10.3390/plants10040678 (PMC8066573; doi:10.3390/plants10040678)

## Slide 1
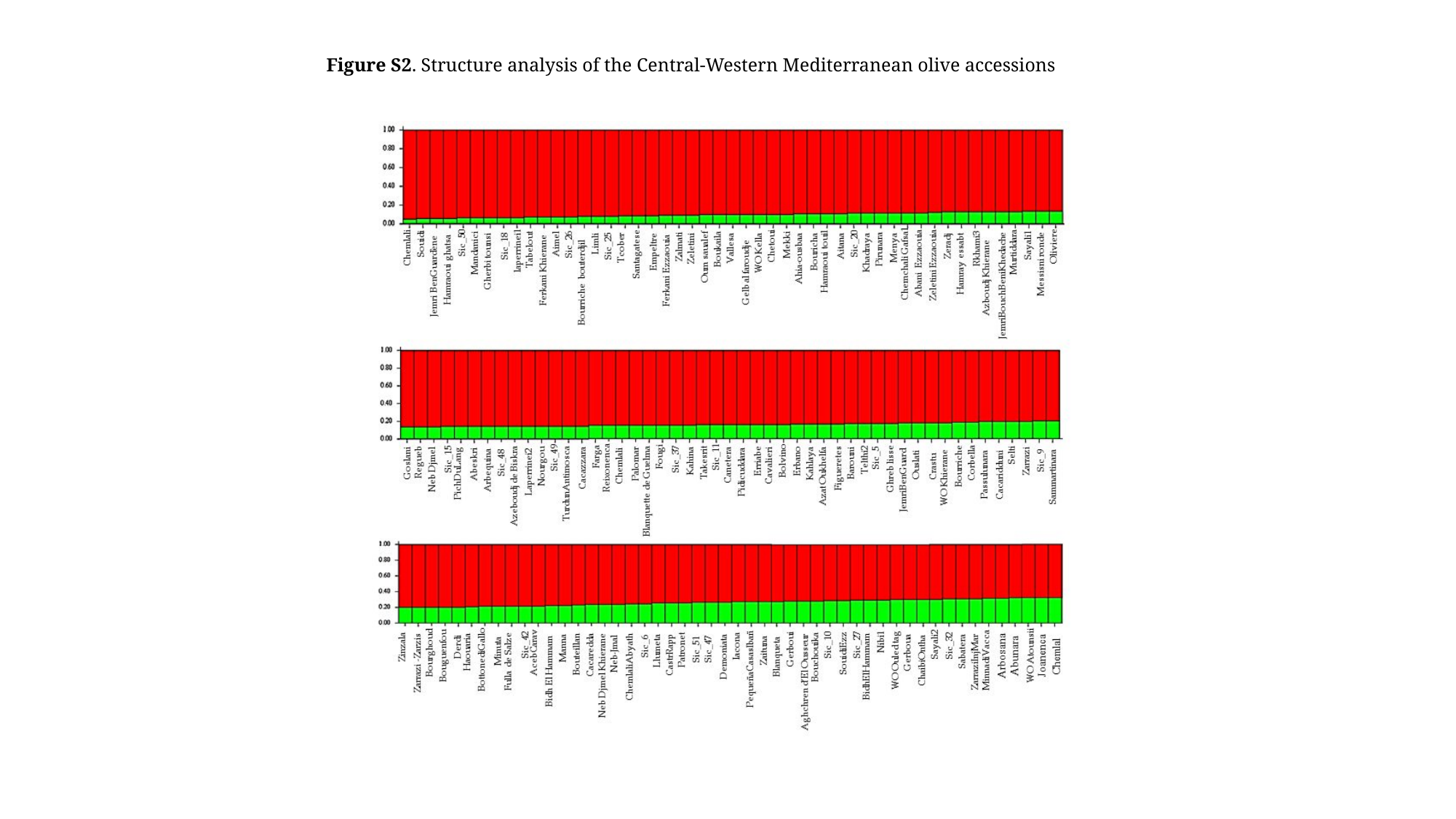

Figure S2. Structure analysis of the Central-Western Mediterranean olive accessions

## Slide 2
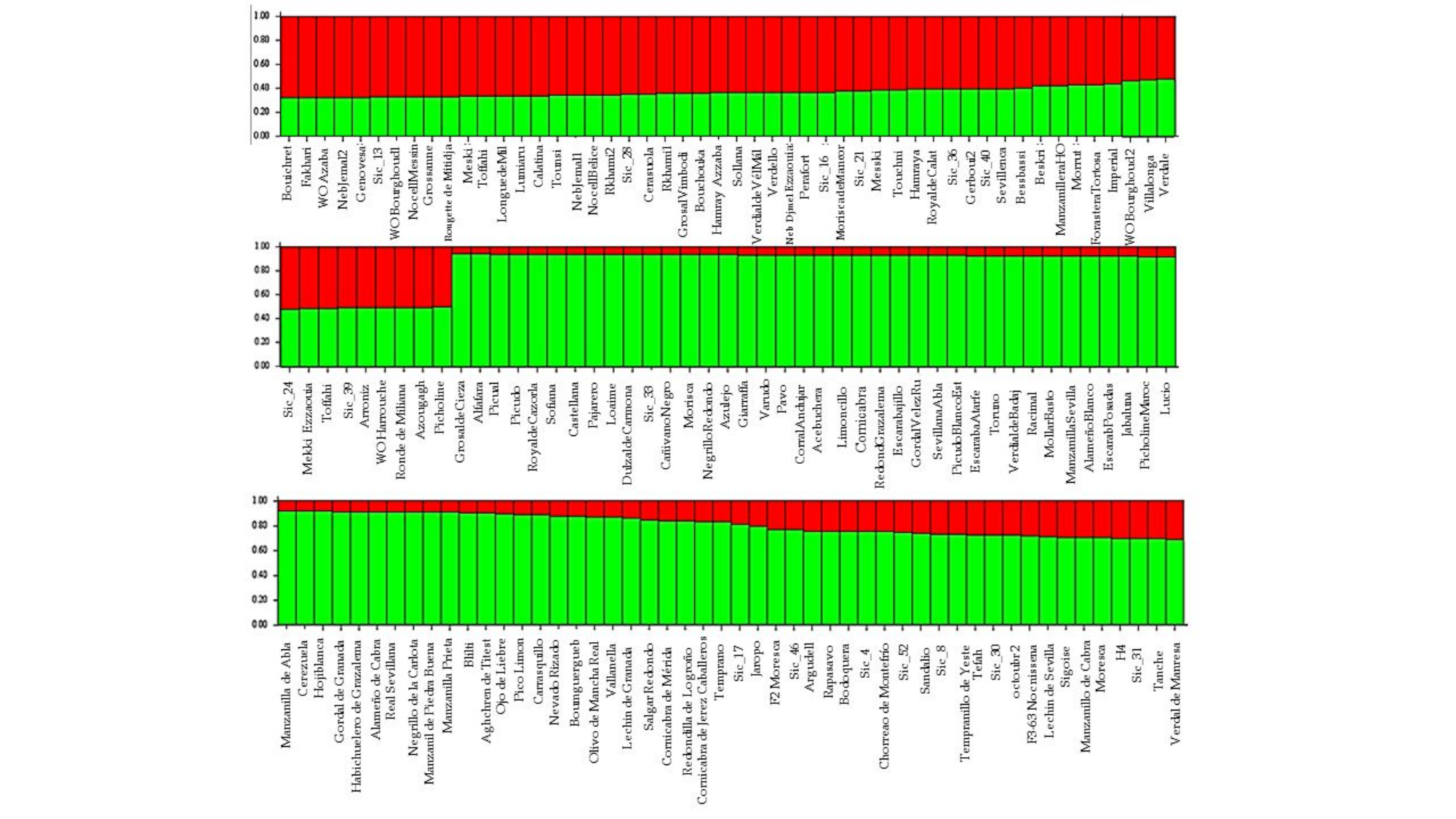

## Slide 3
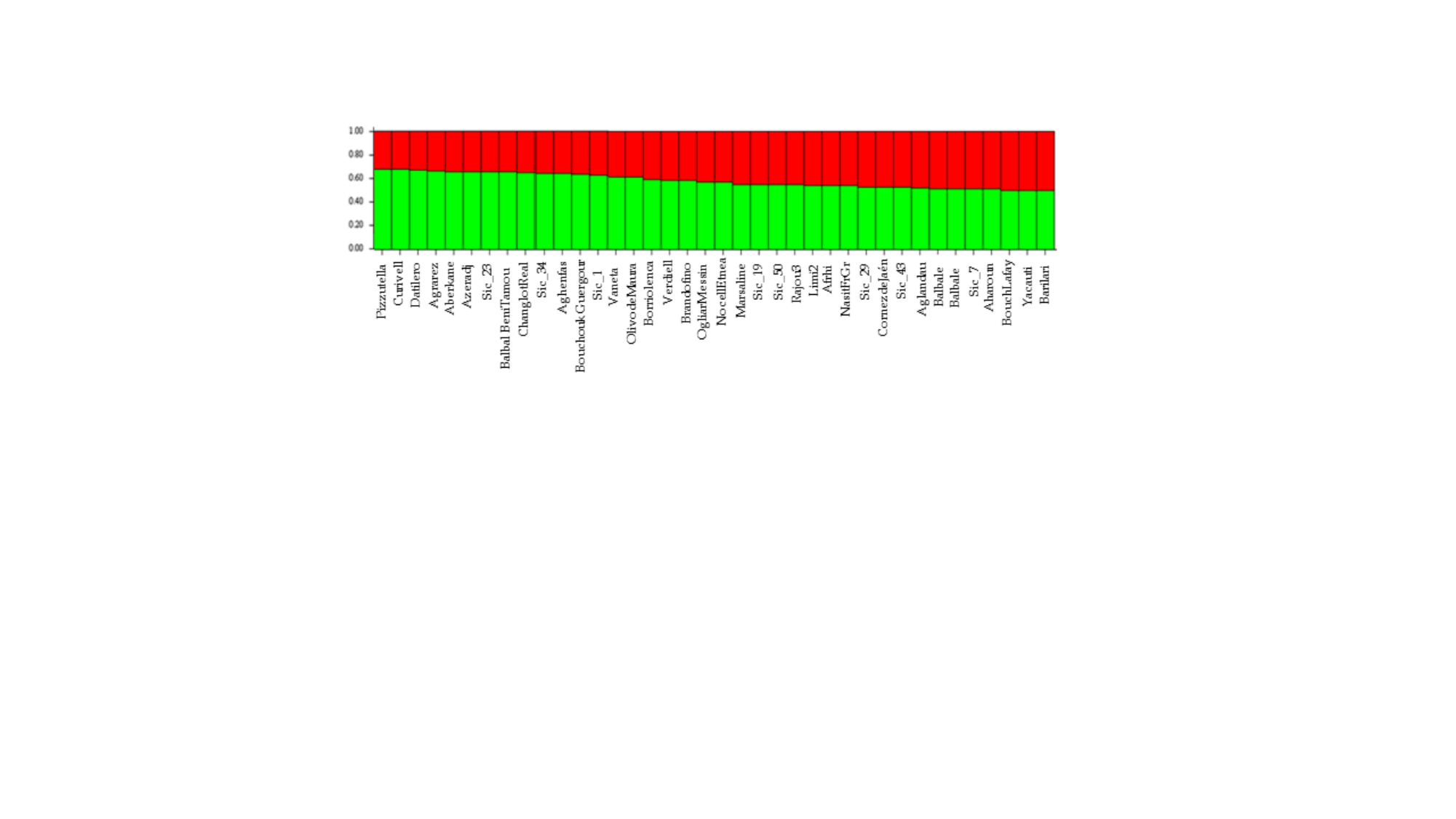

Supplement: Supplementary file 1 [file plants-10-00678-s001.zip › SupplementaryMaterial/FigureS2.pptx]
